# Distribution of Matrix Metalloproteinases in Human Atherosclerotic Carotid Plaques and Their Production by Smooth Muscle Cells and Macrophage Subsets

**DOI:** 10.1007/s11307-015-0882-0

**Published:** 2015-09-16

**Authors:** Nynke A. Jager, Bastiaan M. Wallis de Vries, Jan-Luuk Hillebrands, Niels J. Harlaar, René A. Tio, Riemer H. J. A. Slart, Gooitzen M. van Dam, Hendrikus H. Boersma, Clark J. Zeebregts, Johanna Westra

**Affiliations:** Departments of Rheumatology and Clinical Immunology, University Medical Center Groningen, University of Groningen, PB 30.001, 9700 RB Groningen, The Netherlands; Department of Surgery, Division of Vascular Surgery, University Medical Center Groningen, University of Groningen, Groningen, PB 30.001, 9700 RB Groningen, The Netherlands; Departments of Pathology and Medical Biology, University Medical Center Groningen, University of Groningen, PB 30.001, 9700 RB Groningen, The Netherlands; Department of Cardiology, University Medical Center Groningen, University of Groningen, PB 30.001, 9700 RB Groningen, The Netherlands; Departments of Nuclear Medicine and Molecular Imaging, University Medical Center Groningen, University of Groningen, PB 30.001, 9700 RB Groningen, The Netherlands; Departments of Clinical Pharmacy and Pharmacology, University Medical Center Groningen, University of Groningen, PB 30.001, 9700 RB Groningen, The Netherlands

**Keywords:** Matrix metalloproteinase, Macrophage, Atherosclerotic plaque, Smooth muscle cell, MMPSense

## Abstract

**Purpose:**

In this study, the potential of matrix metalloproteinase (MMP) sense for detection of atherosclerotic plaque instability was explored. Secondly, expression of MMPs by macrophage subtypes and smooth muscle cells (SMCs) was investigated.

**Procedures:**

Twenty-three consecutive plaques removed during carotid endarterectomy were incubated in MMPSense™ 680 and imaged with IVIS® Spectrum. mRNA levels of MMPs, macrophage markers, and SMCs were determined in plaque specimens, and in in vitro differentiated M1 and M2 macrophages.

**Results:**

There was a significant difference between autofluorescence signals and MMPSense signals, both on the intraluminal and extraluminal sides of plaques. MMP-9 and CD68 messenger RNA (mRNA) expression was higher in hot spots, whereas MMP-2 and αSMA expression was higher in cold spots. In vitro M2 macrophages had higher mRNA expression of MMP-1, MMP-9, MMP-12, and TIMP-1 compared to M1 macrophages.

**Conclusion:**

MMP-9 is most dominantly MMP present in atherosclerotic plaques and is produced by M2 rather than M1 macrophages.

## Introduction

Atherosclerosis is a progressive inflammatory disease characterized by the accumulation of lipid-filled macrophages within the arterial intima. Continued inflammation may promote rupture of the atherosclerotic plaque’s protective fibrous cap causing subsequent clinical ischemic events [1, 2]. The fibrous cap covering an advanced atherosclerotic plaque is typically composed of smooth muscle cells (SMCs) and extracellular matrix (ECM) [3]. Activated monocyte-derived macrophages and SMCs are critically involved in the development of high-risk vulnerable plaques by producing matrix metalloproteinases (MMPs) [1, 4, 5].

A heterogeneous population of macrophages exists including a classically activated macrophage type (M1) as well as an alternatively activated macrophage population (M2) [6]. The M1 macrophage is thought to have pro-inflammatory properties, and polarization in vitro is driven by low concentration lipopolysacharride (LPS) and interferon-gamma (IFN-γ). Defined as classically, however, M2 macrophages are anti-inflammatory and immune regulatory. Upon cytokine stimulation, they modify development of atherosclerotic plaques. The M2 macrophage population can be divided in M2a, M2b, and M2c subtypes, depending on the cytokine environment (IL-4, immune complexes, and IL-10, respectively)[7]. In contrast to its classical M2 properties, the function of M2a macrophages is type II inflammation, and the function of type M2c is matrix deposition and tissue remodeling; this last mentioned type might be most important in atherosclerosis [8]. Recently, Wolfs et al. suggested that additional circumstances in the local microenvironment makes macrophage polarization in the atherosclerotic tissue even more complex than the typically described M1 and M2 macrophage distribution [9]. So, not only cytokine environment, but also foam cell formation and chemokine ligand 4 (CXCL4), among other factors, play major roles.

MMPs are proteolytic enzymes that can degrade ECM proteins such as gelatin (MMP-2 and MMP-9), collagen (MMP-1, MMP-8, and MMP-13), elastin (MMP-12), and fibrin (MMP-3 and MMP-10). They can be inhibited by tissue inhibitors of metalloproteinases (TIMPs). The ever-growing MMP family now consists of more than 20 known proteins [10]. There is conflicting evidence about the role of MMP-9 in atherosclerosis. In carotid atherosclerotic disease, MMP-9 is associated with the development of unstable plaques. In patients with symptomatic carotid disease, increased MMP-9 levels have been shown both in plasma and in plaque tissue [11–16]. Other studies showed an inverse relation between plaque activity and MMP-9 plasma levels or even evidence for a plaque-stabilizing role for MMP-9 [17, 18]. At the moment, MMPs cannot be visualized with conventional imaging modalities, such as duplex ultrasound, computerized tomography scanning, and magnetic resonance imaging. Although these conventional imaging techniques have improved and do have the ability to image cardiovascular anatomy and physiology on a macroscopic scale, they lack the possibility to detect biological processes at the cellular or molecular level [19]. In contrast, molecular imaging has the possibility to target molecular components of atherosclerotic disease on a microscopic scale using smart activatable probes [20]. As such, MMPs may be targeted with a MMP-sensitive probe (MMPSense) and can be visualized by fluorescence imaging [21–23]. MMPSense is a protease activatable fluorescent imaging agent that is activated by MMP-2, MMP-3, MMP-9, and MMP-13. MMPSense is optically silent in its inactivated state and becomes highly fluorescent following protease-mediated activation. Previously, we showed that MMPSense detected most likely MMP-9 in hot spots, which are in regions with high uptake [23]. Also, increased presence of CD68-positive macrophages was present in these hot spots.

In this study, we analyzed the presence of MMPs in human carotid plaques by using MMPSense and investigated differences in intensity of fluorescence signals. Furthermore, monocytes were in vitro differentiated and polarized into M1, M2a, and M2c macrophages to investigate expression of MMPs in these subtypes, as well as in SMCs. In this way, the potential of MMPSense as marker for atherosclerotic carotid plaque vulnerability was explored, and the relation to MMP expression of macrophage subtypes and SMCs throughout atherosclerotic plaques.

## Materials and Methods

### Study Design

Patients presenting with symptoms (i.e., with a history of recent cerebrovascular accident (CVA)), transient ischemic attack (TIA or amaurosis fugax), and a stenosis of the common carotid artery of 70–99 % as detected by duplex ultrasound examination underwent open carotid surgery at the University Medical Center Groningen (UMCG). Additionally, asymptomatic patients (mean age 71 years, range 51–81 years) with a stenosis of 80–99 %, found by routine control were also eligible for surgical treatment. Therefore, a total of 23 carotid specimens were obtained by means of carotid endarterectomy (CEA) of the carotid bifurcation using standard techniques [24]. Risk factors such as hyperlipidemia, hypertension, smoking status, obesity (body mass index (BMI)), and diabetes mellitus were recorded. Hyperlipidemia and hypertension were defined as described before by our group [25]. To measure MMP expression in differentiated macrophages, ten healthy volunteers were included without known cardiovascular disease or risk factors. The study was approved by the Institutional Review Board (IRB) of the UMCG, and informed consent was obtained from all individual participants included in the study.

### Carotid Endarterectomy Sample Collection and Timepath of Study

Plaques were obtained immediately after endarterectomy. Following endarterectomy, all specimens were washed with PBS to remove blood and debris. After storage in PBS, samples were put on ice and taken to the laboratory for fluorescence imaging. The plaques were cut open longitudinally and pinched on a silicone plate with nonreflective black paper (XPB-24 black paper, Caliper Life Sciences, Hopkinton, MA, USA) in between to reduce autofluorescence. Then, autofluorescence signals were recorded on the intraluminal and extraluminal sides of the plaque. The mean time between excision of the plaque and determination of autofluorescence was 31 min (16–45). It took 9 min (2–26) to complete the autofluorescence recordings. After that, the plaque was incubated with a MMP-sensitive activatable fluorescent probe (MMPSense^™^680, VisEn Medical, Boston, MA, USA), which was diluted 1:10. Specimens were incubated for 66 min (60–72) before imaging.

### Fluorescence Imaging and Data Analysis

Fluorescence images were obtained with a commercial imaging system with an ultra-sensitive charge-coupled device camera mounted on a light-tight black chamber (IVIS^®^ spectrum, Caliper Life Sciences, Hopkinton, MA, USA). The charged-coupled device (CCD) camera was cooled to −90 °C. The excitation and emission filter were set at Cy5.5. By dividing the mean radiance (p/s/cm^2^/sr) from the MMPSense signal by the autofluorescence signal, the target to background ratio (TBR) was calculated. The imaging data were analyzed using Living Image^®^ 3.0 software (Caliper Life Sciences, Hopkinton, MA, USA).

### Immunohistochemistry

Slices of plaques were embedded in paraffin, and sections of 4 μm were cut. Macrophages were identified by incubation with monoclonal mouse anti-human CD68 (1:50; mo876 clone PG-M1 DAKO, Glostrup, Denmark). For detection of MMP-9, a goat anti-human antibody (AB911, R&D systems, Minneapolis, USA) was used. Appropriate secondary antibodies labeled with horseradish peroxidase (HRP) were used. Color reaction was developed using DAB staining with chromogen, and sections were counterstained with hematoxylin.

### In Vitro Differentiation and Polarization of M1, M2a, and M2c Macrophages

From ten healthy donors peripheral blood mononuclear cells (PBMCs) were isolated by density gradient centrifugation using Lymphoprep (Axis Shield PoC As, Oslo, Norway). Subsequently, monocytes were allowed to adhere to culture plates. The adherent cells were maintained for 5 days in RPMI 1640 medium (Lonza, Walkersville, MD, USA) supplemented with 10 % filtered fetal calf serum (FCS) and 20 ng/ml macrophage colony-stimulating factor (M-CSF, R&D Systems) for differentiation into macrophages. Macrophages were directed toward M1, M2a, and M2c phenotypes by use of 48-h stimulation with 100 U/ml IFN-γ (PeproTech, USA) and 1 ng/ml LPS (Sigma, Germany) (leading to M1), 20 ng/ml IL-4 (M2a), or 10 ng/ml IL-10 (M2c) (PeproTech) or both IL-4 and IL-10 (IL-4/IL-10), respectively. Validation of M1 and M2 marker expression after differentiation was shown previously [26]. In that paper, we showed that in vitro differentiated M1 macrophages have a higher messenger RNA (mRNA) expression of pro-inflammatory TNF-α and TLR-2 compared to M2 macrophages (IL-4/IL-10), who in turn have higher mannose receptor and IL-10 mRNA expression. On receptor level, M1 showed to have higher CD86 expression, while M2 macrophages have a higher CD163 expression. By this method of differentiation indeed M1 and M2 markers are expressed on these macrophages. MMP-9 protein was measured in supernatants of cultured macrophages with ELISA (duoset, R&D Systems) according to manufacturer’s description.

### RNA Expression In Vitro in Macrophage Subtypes and SMCs and Ex Vivo in Plaques

To measure MMP expression, RNA was extracted from above-named macrophage subtypes and from SMCs as described before [25]. From four imaged plaques, areas with high intensity (hot spots) and low intensity (cold spots) were excised and mRNA was isolated from these specimens. Four un-imaged plaques were divided in equal slices of 5 mm, and mRNA was also isolated from these slices. Complementary DNA (cDNA) samples were added in duplicate for amplification by the TaqMan real-time PCR system (ABI Prism 7900HT Sequence Detection system, Applied Biosystems, Foster City, CA, USA). mRNA expression of MMP-1, MMP-2, MMP-3, MMP-8, MMP-9, MMP-12, MMP-13, MMP-14, –MMP-16, and TIMP-1 was measured by using TaqMan primer/probes sets. The TaqMan primer/probe sets were the following: GAPDH: Hs9999905_m1; MMP1: Hs00233958_m1; MMP2: Hs01548727_m1; MMP3: Hs00233962_m1; MMP8: Hs01029057_m1; MMP9: 00234579_m1; MMP12: Hs00899662_m1; MMP13: Hs00233992_m1; MMP14: Hs01037009_m1; MMP16: Hs00234676_m1; and TIMP-1: Hs00171558_m1. In slices of plaques, also mRNA expression of CD68 (pan macrophages), CD86 (M1 macrophage marker), CD163 (M2 macrophage marker), and αSMA (ACTA2) was investigated. Threshold cycle (Ct) values were determined using the software program SDS 2.4 (Applied Biosystems). Relative gene expression was normalized to the expression of glyceraldehydes-3-phosphate dehydrogenase (GAPDH) and calculated by the following formula: relative gene expression = 2^–ΔCt^ (ΔCt = Ct gene of interest − Ct GAPDH).

### Statistical Analysis

Values are presented as mean ± standard deviation or median (range), unless stated otherwise. For correlations, Pearson’s and Spearman’s correlation coefficients were used when appropriate. A two-tailed, paired Student’s *t* test was used for parametric distributions (i.e., fluorescence signal and TBR). Nonpaired continuous variables with a nonparametric distribution were analyzed using the Mann-Whitney *U* test or with a Wilcoxon test in case of paired samples. For comparing more than two independent samples, the Kruskal-Wallis test (ANOVA) was used (i.e., four types of macrophages). A two-sided *p* value <0.05 was considered statistically significant. Statistical tests were done with the Statistical Package for the Social Sciences (SPSS statistics version 20.0, SPSS Inc.^®^, Chicago, IL, USA).

## Results

### Patient Demographics

A total of 15 men and eight women with a mean age of 70 ± 9 years were included. Baseline characteristics of participants are shown in Table 1.

**Table 1:** Baseline characteristics and risk factors for atherosclerosis

	Patients (*n* = 23)
Men, *n* (%)	15 (65)
Age (years)	70 ± 9
Symptomatic, *n* (%)	21 (91)
Transient ischemic attack (TIA), *n* (%)	9 (39)
Cerebro vascular accident (CVA), *n* (%)	8 (35)
Amaurosis fugax, *n* (%)	4 (17)
BMI (kg/m^2^)	27 ± 3
Smoking status, *n* (%)	10 (43)
>1 pack a day, *n* (%)	6 (26)
≤1 pack a day, *n* (%)	4 (17)
None, smoked in last 10 years (%)	6 (26)
Hypertension, *n* (%)	19 (83)
Controlled with single drug, *n* (%)	5 (21)
Controlled with two drugs, *n* (%)	5 (21)
Requires >2 drugs or uncontrolled, *n* (%)	9 (39)
Systolic blood pressure (mmHg)	146 ± 22
Diastolic blood pressure (mmHg)	80 ± 12
Hyperlipidemia, *n* (%)	14 (61)
Use of lipid-lowering drugs, *n* (%)	9 (39)
Diabetes mellitus, *n* (%)	7 (30)^a^

^a^Four patients had diabetes controlled by diet or oral agents; three patients were on insulin. Data are expressed as mean ± standard deviation; percentages are between brackets

### Fluorescence Imaging

**Fig. 1.**
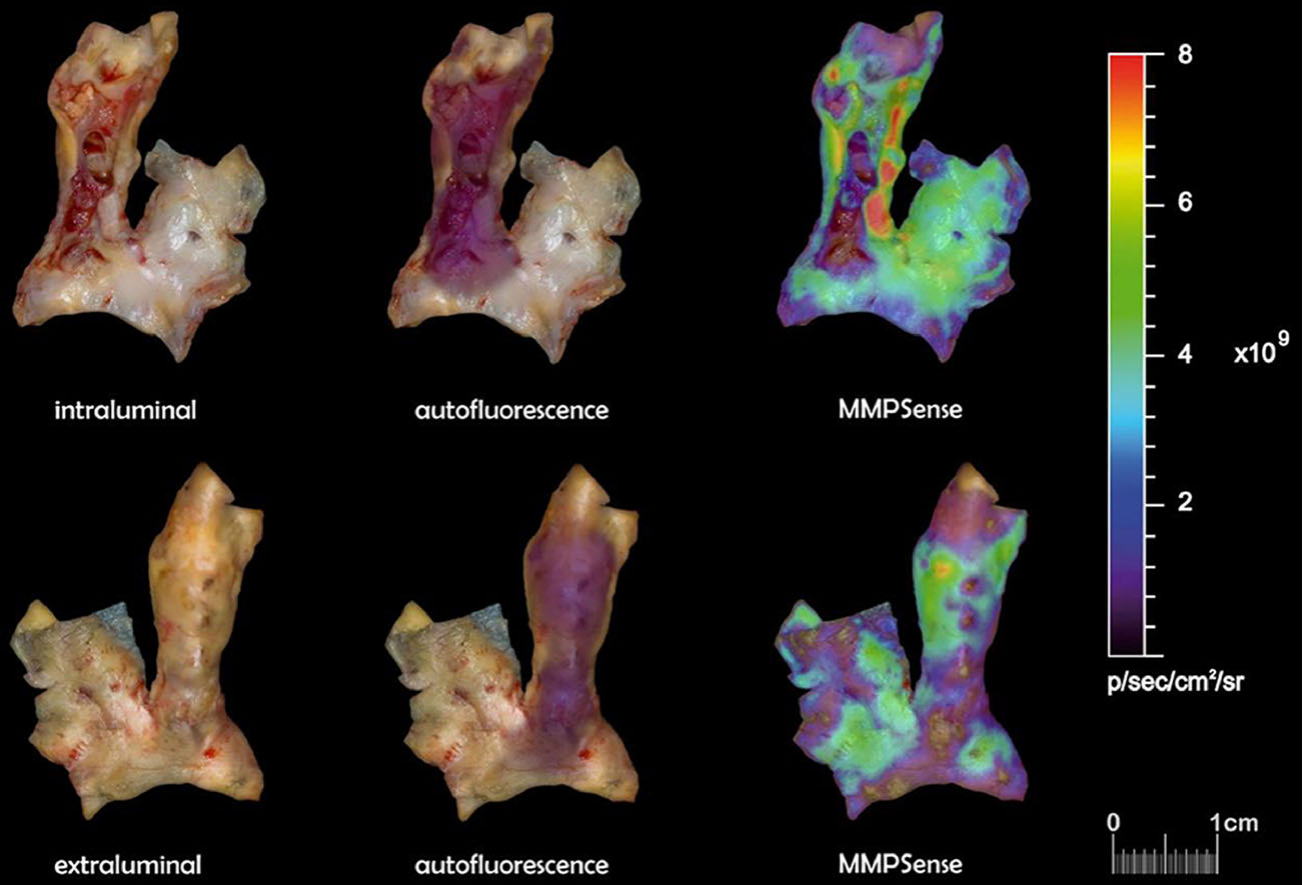
Ex vivo fluorescence imaging of human atherosclerotic plaques. Fluorescence signals reflecting MMPSense activation in specific areas of plaque intraluminally (*upper*) and extraluminally (*bottom*): incubation in phosphate-buffered saline (*left*), autofluorescence (*middle*), and incubation with MMPSense.

**Fig. 2.**
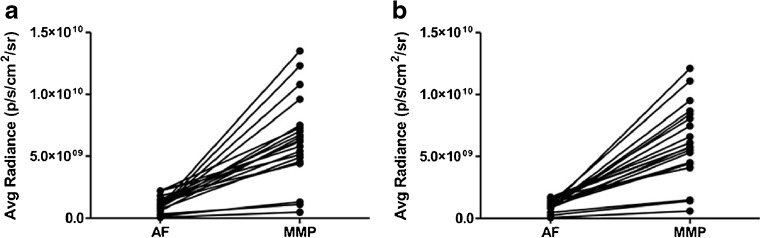
Intensity of fluorescence signals after incubation with MMPSense compared to autofluorescence. Fluorescence signals of plaques incubated with MMPSense (MMP) were increased both **a** intraluminally and **b** extraluminally when compared with autofluorescence (AF). Intraluminal (IL) side (mean value 6.34 × 10^9^ vs 1.09 × 10^9^; *p* < 0.0001) and extraluminal (EL) side (mean value 6.12 × 10^9^ vs 1.04 × 10^9^; *p* < 0.0001).

Fluorescence signal of each plaque was recorded before and after incubation with MMPSense^™^680. MMP signals were heterogeneously distributed throughout plaques (Fig. 1). The mean TBR was considered appropriate and did not significantly differ between intraluminal and extraluminal sides (7.15 vs 6.43; *p* = 0.53). Fluorescence signals clearly augmented after incubation with MMPSense compared to autofluorescence, showing highly significant differences on both intraluminal (mean value 6.34 vs 1.09; *p* < 0.0001) and extraluminal sides (mean value 6.12 vs 1.04; *p* < 0.0001) (Fig. 2).

### Ex Vivo MMP, αSMA, and Macrophage Expression in Plaques

**Fig. 3.**
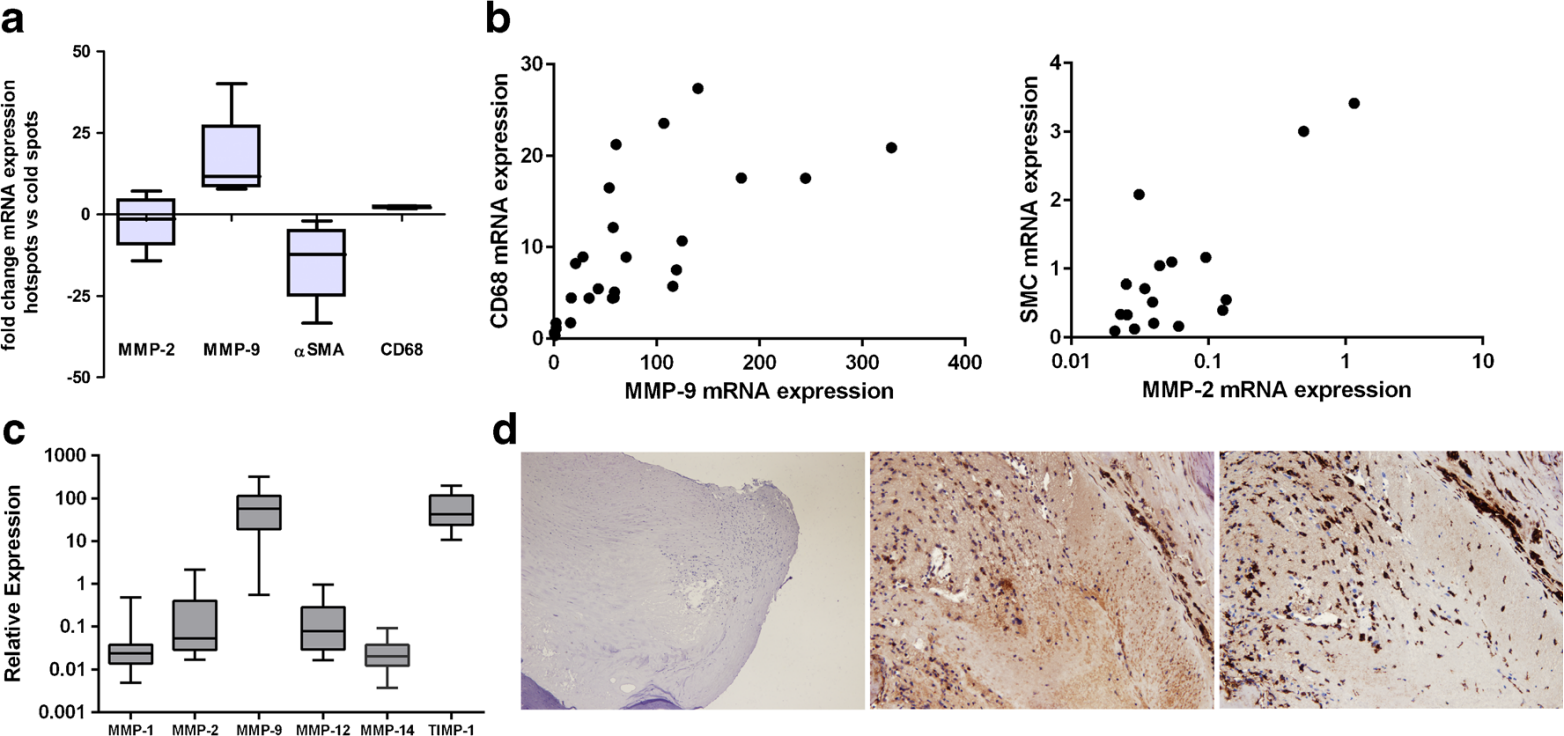
Expression of MMPs in atherosclerotic plaques. Quantitative reverse transcriptase polymerase chain reaction (qRT-PCR) was used to determine relative mRNA expression of different genes in human atherosclerotic plaques. **a** Areas with high intensity (hot spots) and low intensity (cold spots) determined after incubation with MMPSense were excised from four imaged plaques and subjected to RNA isolation and qRT-PCR. mRNA of MMP-2, MMP-9, CD68, and αSMA, expressed as fold change in hot spots versus cold spots, was determined. **b** Relative mRNA expression of MMP-2, MMP-9, αSMA, and CD68 was determined in slices from four unimaged plaques with qRT-PCR. MMP-9 and CD68 mRNA expression was significantly correlated and also MMP-2 and αSMA mRNA expression (Spearman’s correlation). **c** Relative expression levels of different MMPs were determined with qRT-PCR in slices from four unimaged plaques. **d** Immunohistochemical staining of CD68 (*middle panel*) and MMP9 (*right panel*) in a plaque specimen (*left panel*).

MMP-2, MMP-9, αSMA, and CD68 mRNA expression was investigated in hot and cold spots from 4 imaged plaques. As can be seen in Fig. 3a, MMP-9 and CD68 expression was up regulated in hot spots, whereas MMP-2 and αSMA were downregulated in hot spots. To investigate the expression and distribution of MMPs, and their relation to M1 and M2 macrophages in plaques, slices of unimaged plaques (with intact mRNA) were used for mRNA isolation. Expression of MMPs was compared to CD68 (pan macrophages), and to an M1 marker (CD86) and M2 macrophage marker (CD163). Also, MMP expression was compared to αSMA expression. The strongest correlation was found between MMP-9 and CD68 mRNA expression (*ρ* = 0.792, *p* < 0.001, Fig. 3b). Furthermore, MMP-9 expression was 100 to 1000 times higher compared to mRNA expression of other MMPs (Fig. 3c). None of the other MMPs showed a significant correlation with M1 and M2 macrophage markers, except for MMP-2 and MMP-14 which both correlated significantly with CD86 and CD163. There was a significant correlation between MMP-2 and αSMA expression (*ρ* = 0.534, *p* = 0.0027, Fig. 3b). Immunohistochemical staining of plaques for CD68 and MMP-9 showed overlap of CD68 and MMP-9 as can be seen in a representative picture in Fig. 3d.

### MMP Expression in Macrophage Subtypes and SMCs In Vitro

**Fig. 4.**
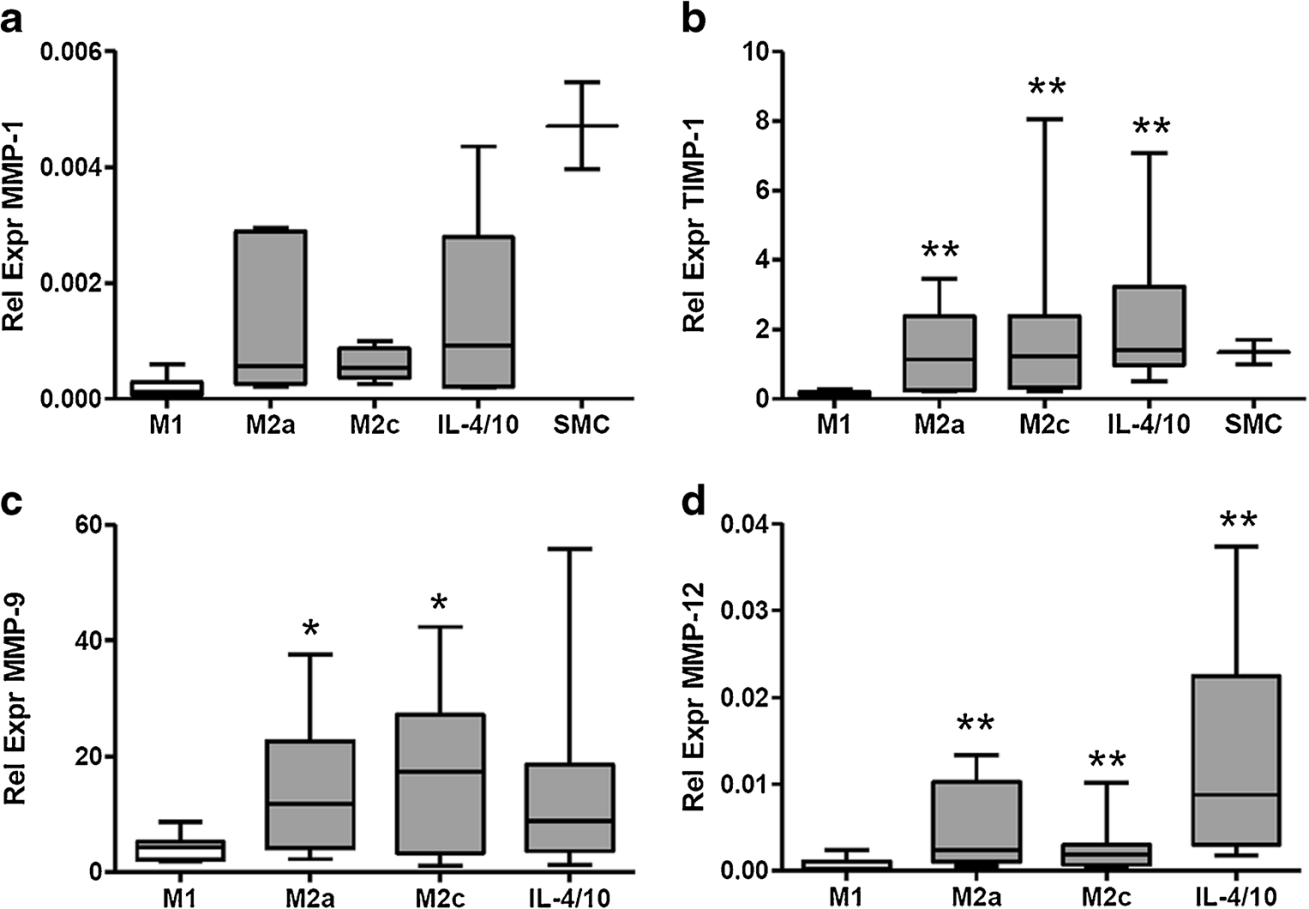
Relative expression of MMP mRNA levels in macrophages from ten healthy volunteers and in SMCs. M1- and M2-polarized macrophages were cultured from monocytes from ten healthy volunteers and subjected to RNA isolation and qRT-PCR. Significantly higher relative expression in M2-like macrophages (*gray bars*) compared to M1-like macrophages (*open bars*) was measured for **a** MMP-1, **b** TIMP-1, and **d** MMP-12. **c** MMP-9 was also higher. Relative expression of MMP-1 and TIMP-1 in SMCs is added in panels **a** and **b**, expression of MMP-9 and MMP-12 was undetectable in SMCs. Significant differences (Wilcoxon paired *t* test) of M2 subtype compared to M1 macrophages is shown in figure: *p < 0.05, **p < 0.01.

**Fig. 5.**
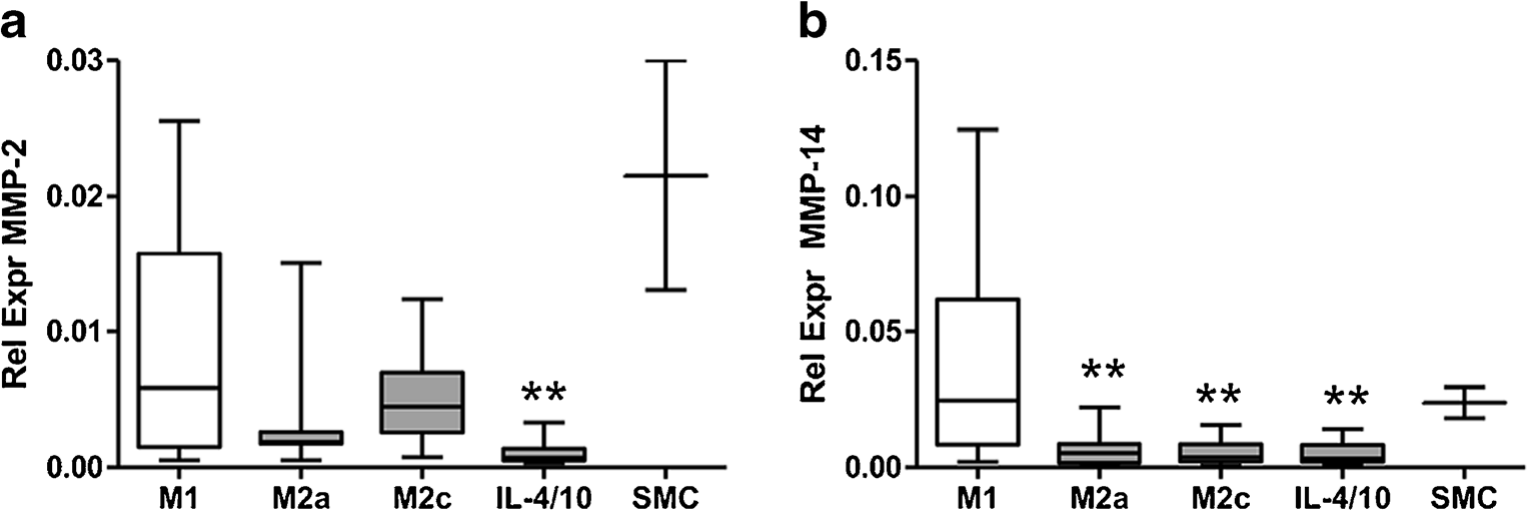
Relative expression of MMP mRNA levels in macrophages from ten healthy volunteers and in SMCs. M1- and M2-polarized macrophages were cultured from monocytes from ten healthy volunteers and subjected to RNA isolation and qRT-PCR. Significantly lower relative expression in M2 macrophages (*gray bars*) compared to M1-like macrophages (*open bars*) was measured for **a** MMP-2 and **b** MMP-14. Significant differences (Wilcoxon paired *t* test) of M2 subtype compared to M1 macrophages is shown in figure: *p < 0.05, **p < 0.01.

**Fig. 6.**
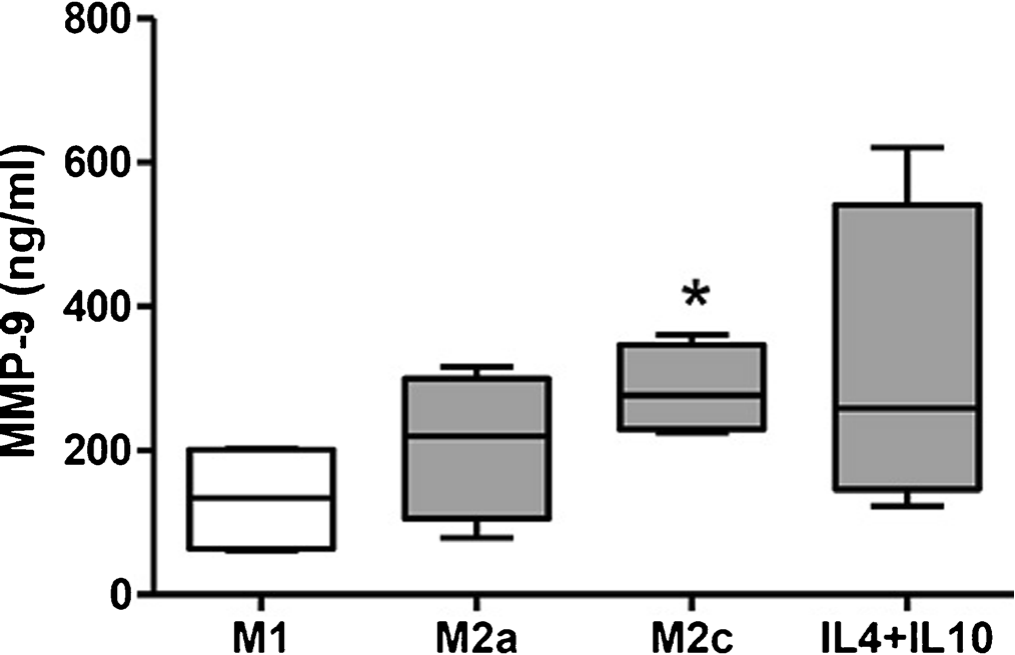
MMP-9 protein levels in supernatants of cultured M1 and M2 macrophages measured by ELISA. M1- and M2-polarized macrophages were cultured from monocytes from ten healthy volunteers, and supernatant was harvested after differentiation. MMP-9 protein levels were measured by ELISA, and these were higher in M2 macrophages (*gray bars*) compared to M1 macrophages (*open bars*). Significant differences (Wilcoxon paired *t* test) of M2 subtype compared to M1 macrophages is shown in figure: *p < 0.05.

mRNA levels of GAPDH (household gene), MMP-1, MMP-2, MMP-3, MMP-8, MMP-9, MMP-12, MMP-13, MMP-14, MMP-16, and TIMP-1 were determined in M1, M2a, M2c, and IL-4/IL-10-differentiated M2 macrophages from ten healthy volunteers. mRNA expression of MMP-1 was significantly increased in all three M2 macrophage types compared to M1 macrophages (*p* = 0.0395 (Kruskall-Wallis (KW)), Fig. 4a). TIMP-1 expression was significantly decreased in M1 macrophages compared to all M2 macrophages (*p* = 0.0002 (KW), Fig. 4b). MMP-9 and MMP-12 mRNA was undetectable in SMCs. MMP-9 expression was higher but not significantly increased in M2 macrophages compared to M1 (ns, KW; Fig. 4c). However, the difference between M1 and M2a or M2c macrophages was statistically significant (Wilcoxon paired *t* test, *p* < 0.05, Fig. 3c). MMP-12 was significantly higher expressed in M2 macrophages compared to M1 macrophages (*p* = 0.0004 (KW), Fig. 4d). On the contrary, MMP-2 and MMP-14 were significantly higher in M1 macrophages compared to all types of M2 macrophages (*p* = 0.0047, *p* = 0.0165, respectively (KW), Fig. 5). Of note, MMP-1 and MMP-2 expression was high in SMCs. MMP-3, MMP-8, MMP-13, and MMP-16 were undetectable in in vitro generated macrophages and in SMCs. From mRNA data in plaques, it was shown that expression of MMP-9 was 100 to 1000 times higher compared to mRNA expression of other MMPs (Fig. 3c). Therefore MMP-9 protein secretion was investigated in supernatants of cultured macrophages. As can be seen in Fig. 6, all different types of macrophages can produce MMP-9 although M2 macrophages produce more than M1 (ns when tested with KW test, *p* = 0.029, paired *t* test for M1 against M2c). So protein levels of MMP-9 are comparable to the mRNA expression of MMP-9 in different subtypes of macrophages.

## Discussion

Our study shows that fluorescence imaging with a smart MMP-sensitive activatable probe clearly reveals a heterogeneous distribution of MMPs across the atherosclerotic carotid plaques. The signals of ex vivo human carotid plaques were significantly enhanced after incubation with the fluorescent probe accounting for a 6- to 7-fold increase of signals. MMP-9 mRNA was found to be highly expressed in plaques and in different subtypes of M2 macrophages.

Signal enhancements such as with MMPSense have been described using other protease probes, both in ex vivo carotid specimens [22], and in vivo rabbits [27]. Typically, there are more intense signals (hot spots) near the carotid bifurcation. In a previous study, segments at or near the bifurcation and segments with intraplaque hemorrhage contained higher MMP levels and activity (especially MMP-9) compared to segments distant from the bifurcation. Histologically stable plaques contained lesser amounts of MMPs, which were predominantly MMP-2. TIMPs were highly abundant in fibrotic and necrotic segments [28]. In the present study, it was shown that using MMPSense in atherosclerotic plaques, mRNA expression of MMP-9 was found to be increased in areas with high intensity (hot spots) compared to areas with low intensity (cold spots) and accompanied with a slight increase in CD68 mRNA expression, as was previously found by Wallis-de Vries et al. [23]. In the latter study, also increased enzymatic gelatinase activity was shown in hot spots, and this was appointed to increased presence of active MMP-9. mRNA expression of MMP-2 was decreased in hot spots compared to cold spots, and also αSMA expression. Also, good correlations in plaques between mRNA expression of MMP-9 and CD68, and between MMP-2 and SMCs were found. These data are supported by another study, where macrophage-rich lesions showed higher MMP-9 activity while SMC-rich lesions showed higher MMP-2 activity [29]. Also, SMCs were found in stable plaques in other studies [29, 30]. However, in an animal study using the mouse brachiocephalic artery model of plaque instability, apolipoproteinE (apoE) knockout mice were crossed with MMP-3, MMP-7, MMP-9, or MMP-12 knockouts [18]. Johnson et al. found that in the apoE/MMP-3 and apoE/MMP-9 double knockouts brachiocephalic artery plaques were significantly larger than in controls and had reduced macrophage content. They concluded that MMP-3 and MMP-9 normally played protective roles, promoting stable plaques. It is unclear to what extent this animal model can be compared to the human situation.

In the present study, MMP-9 mRNA is abundantly present in plaques and highly expressed by macrophages. In a study by Loftus et al., the character, level, and expression of MMPs in carotid plaques was correlated to clinical status of patients undergoing carotid endarterectomy. The MMP-9 concentration was significantly higher in patients developing symptoms within 1 month compared to asymptomatic patients [11]. Also, in a study done by Heo et al., plaque rupture was significantly associated with the development of vascular events in carotid atherosclerotic disease and with immunohistochemical expression of MMP-2 and MMP-9 [31]. One explanation for this might be that MMP-2 and MMP-9 are capable of degrading collagen type IV which is the major component of the basement membrane [14]. No difference in the levels of MMP-1, MMP-2, or MMP-3 was found between symptomatic and asymptomatic patients [11]. It was also anticipated that serum levels of MMP-9 and MMP-2 were significantly higher in symptomatic patients compared to patients without symptoms [12, 13]. However, another group found serum MMPs where not predictive of local events, in a group of 18 patients [32]. In the present in vitro study, mRNA expression of MMP-2 was highest in pro-inflammatory M1 macrophages and in SMCs. MMP-9 and MMP-12 mRNA was highest in M2 macrophages and could not be found in SMCs. This was supported by other groups who found an overexpression of MMP-9 in M2 macrophages [33], and differentiation toward M2 macrophages upregulated MMP-12 expression [4]. However, Newby et al. also suggest that each macrophage subtype (not only M2) can be acted on by pro-inflammatory mediators leading to activated states [4]. Further research is needed to fully understand the mechanism of MMPs produced by macrophages in the process of an atherosclerotic plaque becoming vulnerable. Applications for noninvasive optical imaging of fluorescent signals could be of less clinical value in coronary atherosclerosis because of the limited penetration depth of only a few millimeters. Therefore, we started testing a radiolabeled MMP tracer in ex vivo atherosclerotic plaques recently, from which the results look promising.

We found MMP-9 mRNA and protein expression was 100 to 1000 times higher compared to other MMPs. Taken everything into account, it seems that MMPsense can be used to detect areas of plaque instability primarily by detection of MMP-9 produced by M2 macrophages.

## Conclusion

It is feasible to image MMP-9 in atherosclerotic tissue ex vivo using a smart activatable fluorescence probe and fluorescence imaging. MMP-9 is produced by macrophages and is abundantly present in plaques as shown by immunohistochemical staining and qRT-PCR. Furthermore, areas with high intensity (hot spots) have a higher mRNA expression of MMP-9 compared to areas with low intensity (cold spots). Also, MMP-9 expression was highest in M2 macrophages and could not be found in SMCs. In conclusion, MMPSense can be used to detect MMP-9 in plaques and might therefore be a good marker to investigate plaque instability.

## Abbreviations

BMI: Body mass index
CCD: Charged-coupled device
CEA: Carotid endarterectomy
Ct: Threshold cycle
CVA: Cerebrovascular accident
CXCL4: Chemokine ligand 4
ECM: Extracellular matrix
ELISA: Enzyme-linked immunosorbent assay
FCS: Fetal calf serum
GAPDH: Glyceraldehyde-3-phosphate dehydrogenase
HRP: Horseradish peroxidase
IFN-γ: Interferon-gamma
IL: Interleukin
IRB: International Review Board
LPS: Lipopolysacharride
M-CSF: Macrophage colony-stimulating factor
MMP: Matrix metalloproteinases
mRNA: Messenger ribonucleic acid
PBMCs: Peripheral blood mononuclear cells
SMCs: Smooth muscle cells
TBR: Target to background
TIA: Transient ischemic attack
TIMP: Tissue inhibitors of metalloproteinases
